# Effect of Chitosan on the Number of *Streptococcus mutans* in Saliva: A Meta-Analysis and Systematic Review

**DOI:** 10.3390/ijms242015270

**Published:** 2023-10-17

**Authors:** Virág Róna, Bulcsú Bencze, Kata Kelemen, Dániel Végh, Réka Tóth, Tamás Kói, Péter Hegyi, Gábor Varga, Noémi Katinka Rózsa, Zoltán Géczi

**Affiliations:** 1 Department of Prosthodontics, Semmelweis University, 1085 Budapest, Hungary; rona.virag@semmelweis.hu (V.R.); bencze.bulcsu@semmelweis.hu (B.B.); kelemen.kata@semmelweis.hu (K.K.); vegh.daniel@semmelweis.hu (D.V.); 2 Centre for Translational Medicine, Semmelweis University, 1085 Budapest, Hungary; rekatoth83@gmail.com (R.T.); samatiok@gmail.com (T.K.); hegyi.peter@semmelweis.hu (P.H.); varga.gabor@semmelweis.hu (G.V.); rozsa.noemi@semmelweis.hu (N.K.R.); 3 Department of Stochastics, Institute of Mathematics, Budapest University of Technology and Economics, 1111 Budapest, Hungary; 4 Institute for Translational Medicine, Medical School, University of Pécs, 7622 Pécs, Hungary; 5 Institute of Pancreatic Diseases, Semmelweis University, 1085 Budapest, Hungary; 6 Department of Oral Biology, Semmelweis University, 1085 Budapest, Hungary; 7 Department of Pediatric Dentistry and Orthodontics, Semmelweis University, 1085 Budapest, Hungary

**Keywords:** chitosan, dental caries, cariogenic bacteria, *Streptococcus mutans* (*S. mutans*), chewing gum

## Abstract

We conducted a meta-analysis and systematic review to investigate the efficacy of chitosan-containing chewing gums, and to test their inhibitory effects on *Streptococcus mutans*. The systematic search was performed in three databases (Cochrane Library, EMBASE, and PubMed) and included English-language randomized–controlled trials to compare the efficacy of chitosan in reducing the number of *S. mutans*. To assess the certainty of evidence, the GRADE tool was used. Mean differences were calculated with a 95% confidence interval for one outcome: bacterial counts in CFU/mL. The protocol of the study was registered on PROSPERO, registration number CRD42022365006. Articles were downloaded (*n* = 6758) from EMBASE (*n* = 2255), PubMed (*n* = 1516), and Cochrane (*n* = 2987). After the selection process, a total of four articles were included in the qualitative synthesis and three in the quantitative synthesis. Our results show that chitosan reduced the number of bacteria. The difference in mean quantity was −4.68 × 10^5^. The interval of the random-effects model was [−2.15 × 10^6^; 1.21 × 10^6^] and the prediction interval was [1.03 × 10^7^; 9.40 × 10^6^]. The I2 value was 98% (*p* = 0.35), which indicates a high degree of heterogeneity. Chitosan has some antibacterial effects when used as a component of chewing gum, but further studies are needed. It can be a promising antimicrobial agent for prevention.

## 1. Introduction

In recent years, there has been a lot of research into the potential use of natural bioactive substances as effective preventive medicines against oral diseases such as dental caries [1,2,3,4]. During the 1980s and 1990s, antimicrobials and antibiotics such as spiramycin and vancomycin were used against *Streptococcus mutans* (*S. mutans*) to minimize plaque-induced diseases such as dental caries [5,6,7]. We avoid classical antibiotics, which affect certain cellular functions and to which resistance develops more easily. Instead, we use a substance that is directly toxic to bacteria, has limited cytotoxicity and is less likely to develop resistance.

Chitosan is a positively charged, biocompatible, and biodegradable substance that can form pores on the cell membranes of bacteria and cause apoptosis in the bacteria [8,9]. Chitosan is produced by the deacetylation of chitin. Chitin is a long polymeric polysaccharide composed of N-acetylglucosamine molecules and is found in a variety of habitats. It is the primary component of the cell wall of fungi and the exterior skeleton of arthropods [10,11,12,13]. Chitosan has been shown to possess a wide range of pharmacological activities, including immuno-potentiation, antihypertensive, serum cholesterol-lowering, antimicrobial, and wound-healing qualities [11,14,15,16,17]. These biological effects are influenced by a wide variety of molecular weights (MW) and deacetylation levels.

Chitosan has antibacterial capabilities and reacts both with Gram-positive and Gram-negative microorganisms, making it an excellent choice for reducing cariogenic bacteria and preventing dental caries [4]. Dental caries is an infectious condition that begins with acid developed in the dental plaque by bacterial metabolism [18]. Tooth decay is a multimicrobial disease caused by various types of bacteria in the mouth. *S. mutans* is a specific type of bacteria that is commonly associated with dental caries, as it can cause low pH and an exopolysaccharide (EPS)-rich milieu [19]. *S. mutans* is divided into four kind of serotypes: c, e, f, and k. Serotype c is the most frequently found in the oral cavity, with an average prevalence of 70–80%, followed by serotype e, with about 20% [20].

The removal of plaque and microorganisms can be achieved by the mechanical removal of the bacterial biofilm; however, this method alone is not sufficient, and the best and most successful plaque control program is a combination of mechanical and chemical techniques [21]. One of the most common targeting delivery systems against plaque components is chewing gum [22,23,24]. Recent evidence suggests that chewing gum containing chitosan is effective in inhibiting the growth of cariogenic bacteria (total bacteria, total *Streptococci*, *S. mutans*) in saliva [25,26,27]. Because of its intrinsic antibacterial qualities, chitosan, a natural-based biopolymer, has tremendous potential as a primary and secondary preventive agent in dentistry. Furthermore, as compared to modern antibacterial compounds, chitosan has a significantly lower toxicity profile, making it a particularly appealing choice for boosting oral health and avoiding tooth infections.

The antibacterial molecular mechanisms of chitosan against *S. mutans* are diverse, as has been widely explored and documented in the scientific literature. First, its positively charged amino groups interact with negatively charged bacterial cell membranes, weakening their integrity, and increasing permeability. This impairs the cell’s critical activities and causes intracellular component leakage. Second, chitosan can suppress *S. mutans’* metabolic activity by attaching to key enzymes and proteins. This effect leads to decreasing the amount of acid production, which reduces the cariogenic activity of the bacteria. By changing the acidic milieu, it can also diminish the number of other cariogenic bacteria. Furthermore, chitosan has been proven to inhibit biofilm development, preventing other oral cariogenic bacteria from sticking to tooth surfaces and leading to dental cavities [28,29].

However, there has been no meta-analysis of chitosan in the dental field, so our systematic review and meta-analysis is a new contribution to the field. Nevertheless, there have been previous analyses in other fields, such as agriculture, internal medicine, etc. [30,31,32,33].

The aim of our current meta-analysis and systematic review was to investigate the antibacterial effects of chitosan-containing chewing gums, particularly on the amount of *S. mutans*. Our aim is to demonstrate the potential use of chitosan for the reduction of *S. mutans*. Our hypothesis is that chitosan reduces the number of *S. mutans* in the oral cavity.

## 2. Methods

We report the findings of our systematic review and meta-analysis in accordance with the Cochrane Handbook and the PRISMA 2020 guidelines [34]. The protocol of the study was registered on PROSPERO, under registration number CRD42022365006.

### 2.1. Eligibility Criteria

This study included randomized–control trials that investigated the effects of chitosan (population: saliva; intervention: chitosan; comparator: placebo; outcome: number of bacteria (CFU/mL)) on *S. mutans* using samples collected from saliva. The studies included used Mitis-salivarius agar containing 0.2 U/mL bacitracin for the selective culture of *S. mutans*.

### 2.2. Information Sources

Our systematic search was conducted on the 19th of October 2022. The last search was conducted on the 15 April 2023. The following search key was used: (saliva) AND (polycationic polymer* OR chitosan) AND “*S. mutans*” OR *Streptococcus mutans* OR *Streptococcus*.

### 2.3. Selection Process and Data Collection

**Figure 1:**
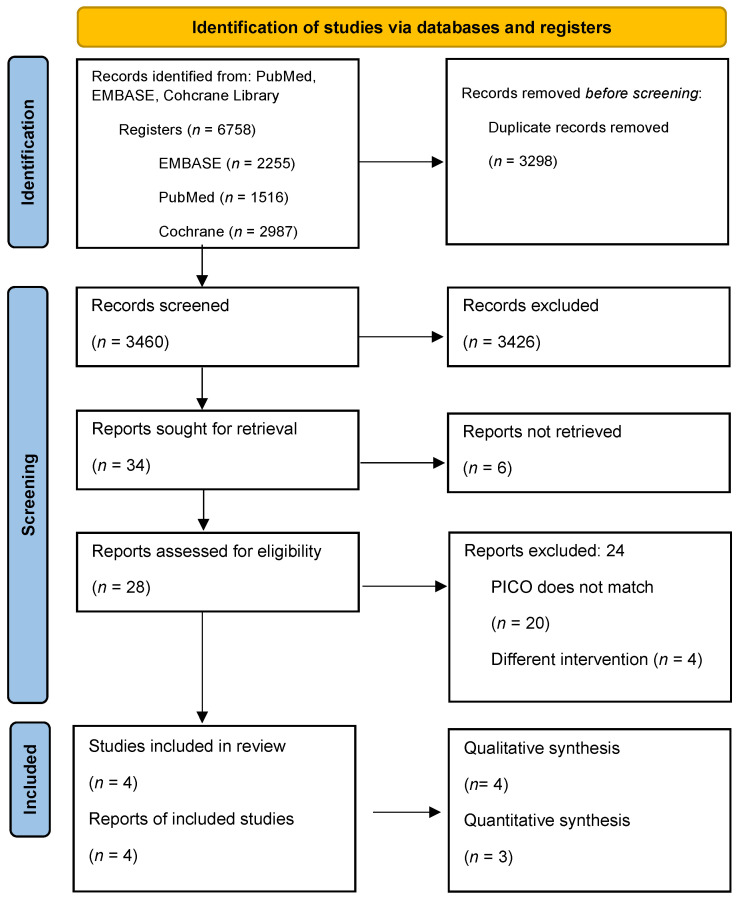
PRISMA 2020 flow diagram for new systematic reviews, which included searches of databases and registers only.

The articles were downloaded from three databases: EMBASE, PubMed, and Cochrane (Figure 1). After duplicate removal, selection was performed by two independent review authors (RV, and BB) using titles and abstracts to select full texts. Data were collected independently from the eligible articles by two authors (RV, and BB). Any disagreements were resolved by the 3rd author (KK). The following information was extracted: first author, publication year, DOI, study type, study population, sample size in the intervention group, and sample size in the comparator group.

### 2.4. Study Risk of Bias Assessment

Two authors (RV, and BB) performed the risk of bias assessment independently, using a Risk of Bias Tool 2 (ROB2) tool [34].

Quality and certainty assessments of the included studies were performed according to the GRADE handbook, using the GRADE-PRO website (https://www.gradepro.org/, accessed on 14 September 2023). The assessment was performed independently by two authors (RV, BB). In cases of disagreement, a third review author was involved (KK).

### 2.5. Synthesis Methods

We extracted or estimated the pre- and post-treatment means and standard deviations, in both the chitosan and control groups. In both groups, we pooled before and after means as well as before–after mean differences in R (v. 4.3.0) using a random-effects model with the inverse variance method and the Hartung–Knapp adjustment. We also pooled the difference of the mean differences. Confidence intervals (CI) of 95% were calculated for the effects of each study and the pooled effects sizes.

Two studies did not report standard deviations (SDs), only means, medians, minima, and maxima. In these cases, the built-in methods of the metacont function were used to estimate SDs. Although the methods provided estimates of the means, we used the available means. In the control group, however, the estimates of means were substantially different from what was reported based on the data [25,26,27]. Hence, the estimated SDs were not used in these cases. Instead, we imputed the reported SDs based on 8 × 10^5^ and 6 × 10^5^ before and after the treatment, respectively [25,26,27]. We also performed a sensitivity analysis with imputed SDs 7 × 10^5^ and 4.5 × 10^5^, yielding similar results.

Another difficulty was the lack of SDs for the before–after changes. Following the advice of the Cochrane Handbook, we estimated correlation coefficients and used these to calculate the SD of change for all three studies [25,26,27]. Sensitivity analyses were conducted with different imputed correlations between 0.7 and 0.95, leading to similar results. In all cases, confidence intervals of 95% were used.

## 3. Results and Discussion

### 3.1. Study Selection

We downloaded 6758 articles from three databases: EMBASE (*n* = 2255), PubMed (*n* = 1516), and Cochrane (*n* = 2987) (Figure 1). After duplicate removal, we had 3460 articles. For full-text selection, 28 abstracts were selected. In total, 4 articles remained at the end of the selection process and 24 articles were excluded because the PICO did not meet our objectives [25,26,27,35].

After the selection process, a total of four articles were included in the qualitative synthesis and three in the quantitative synthesis [25,26,27,35].

### 3.2. Characteristics of the Studies Included

The main characteristics of the studies included in the quantitative analysis are presented in Table 1. All studies were randomized–control trials. In all cases, the intervention group was chitosan, and the control group was placebo. The overall follow-up time in the articles was 30–40 min. In all cases, the investigators collected saliva from the sample group.

### 3.3. Results of Individual Studies and the Synthesis

#### 3.3.1. The Difference of the Mean Differences (CFU/mL) when Using Chitosan and Placebo

**Figure 2:**
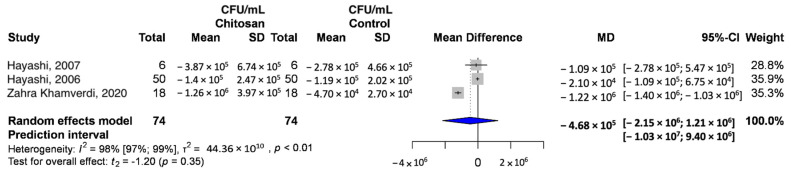
The difference of the mean differences (CFU/mL) when using chitosan and placebo [25,26,27].

Three studies were included in this analysis [25,26,27]. Based on our results, although chitosan reduced bacterial counts in all articles, the reduction was not very noticeable, except in the article by Khamverdi et al., where it was significant. The results for each article were as follows: Hayashi, 2007—MD, −1.09 × 10^5^; 95% CI, [−7.65 × 10^5^; 5.47 × 10^5^]; Hayashi, 2006—MD, −2.1 × 10^4^; 95% CI, [−1.09 × 10^5^; 6.75 × 10^4^]; Khamverdi—MD, −1.22 × 10^6^; 95% CI, [−1.40 × 10^6^; −1.03 × 10^6^]). Only the third article yielded statistically significant differences that were also clinically relevant. According to the results of our meta-analysis, the difference of mean differences was −4.68 × 10^5^. The random-effects model was [−2.15 × 10^6^; 1.21 × 10^6^] and the prediction interval was [−1.03 × 10^7^; 9.40 × 10^6^]. The I^2^ value was 98% (*p* = 0.35), indicating a high degree of heterogeneity amongst the articles (Figure 2). Several serotypes of *S. mutans* may be responsible for the heterogeneity found in our analyzed articles.

#### 3.3.2. Pooled Values of Before–After Control CFU/mL

**Figure 3:**
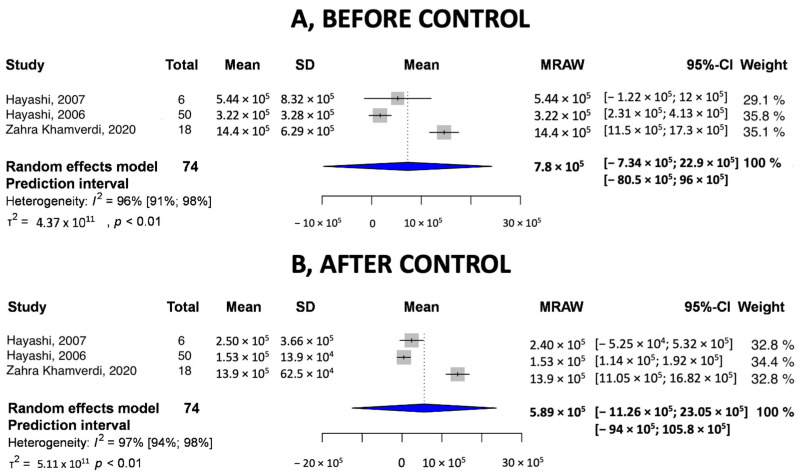
Pooled values of control in CFU/mL (**A**) before control and (**B**) after control [25,26,27].

We compared the pooled values of before–after control groups. Figure 3 shows the mean and standard deviation of the “before control” group. As for heterogeneity, the I^2^ = 96%, and the 95% confidence interval were between −7.34 × 10^5^ and 22.9 × 10^5^ CFU/mL. Figure 3 shows the “after control” pooled values. I^2^ showed 97% heterogeneity, and the 95% confidence interval was between −11.26 × 10^5^ and 23.05 × 10^5^ CFU/mL. We could see that there was a slight decrease in bacterial counts, but no outstanding changes were detected.

#### 3.3.3. Pooled Values of Before–After Intervention CFU/mL

**Figure 4:**
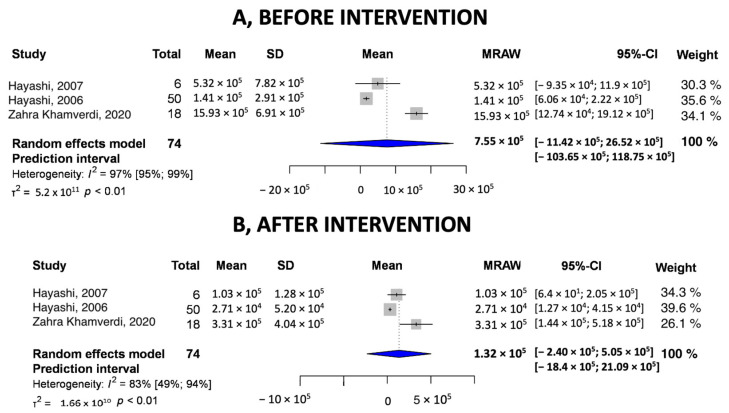
Pooled values of intervention in CFU/mL (**A**) before control and (**B**) after control [25,26,27].

We compared the pooled values of before–after intervention groups. In Figure 4, the I^2^ showed 97% heterogeneity, and the 95% confidence interval was between −11.42 × 10^5^ and 26.52 × 10^5^ CFU/mL. Figure 4 shows the pooled values after chitosan. I^2^ was 83%. The 95% confidence interval was between −2.4 × 10^5^ and 5.05 × 10^5^ CFU/mL. In all cases, bacterial counts were reduced after chitosan use.

#### 3.3.4. Risk of Bias in Studies and Certainty of Evidence

**Figure 5:**
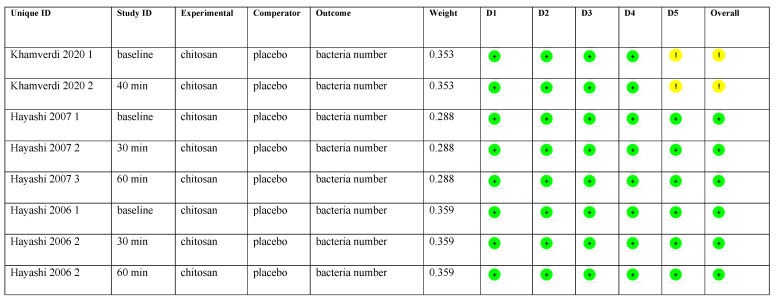
Risk of bias assessment. The meanings of symbols in the figure are green circle = low risk, yellow circle = some concern. D1 to D5 are the domains included in the risk of bias analysis.

We used the ROB2 tool for the risk of bias analysis (Figure 5) [34]. All included articles [25,26,27,35] had a low risk of bias in domains 1–4. In domain 5, bias based on reported results, we considered one of the studies in the article by Khamverdi et al. to be of some concern, because it did not include the prespecified analysis plan, although the authors published the trial protocols.

We found the weight of evidence to be critical, and the risk of bias was not severe (Table S1). PRISMA Checklist can be found in the Supplementary Materials (Table S2).

### 3.4. Discussion

Chitosan, a natural polysaccharide produced from chitin, has received a lot of interest in medicine. Its use has been studied in several medical areas, including drug delivery systems, tissue engineering, and wound covering, making it a promising option for therapeutic treatments [36,37,38,39,40,41,42,43] because of its ability to fight dental caries and cariogenic bacteria such as *S. mutans* [8]. Chitosan employs various approaches to combat dental caries, including its direct antimicrobial activity against cariogenic bacteria. Chitosan causes cell death and inhibits bacterial growth by disrupting the bacterial cell membrane. Chitosan-based mouthwashes and toothpaste formulations have been shown in studies to successfully reduce *S. mutans* and *E. faecalis* counts, reducing the incidence of dental caries [4,8,44,45]. Our meta-analysis suggests that there is a possibility that bacterial counts may be reduced after the use of chitosan. Our pooled data reveal an 80% reduction in bacteria. This was predicted by previous studies to be at least 30% [27]. In our study, we found that chitosan is a great alternative means to reduce the quantity of *S. mutans* in the oral cavity, which is a major contributor to dental caries [19].

Chitosan, a natural biopolymer with antimicrobial properties, has the potential to be used as a primary and secondary preventative approach in dentistry. Because of its low toxicity in comparison to other antimicrobials, chitosan is also a viable choice for dental health and infection prevention [46]. Chitosan nanoparticles loaded with antimicrobial agents, such as chlorhexidine, have shown synergistic effects against cariogenic bacteria, which, according to recent research, results in improved antibacterial activity and the potential for sustained drug release [44].

#### 3.4.1. Molecular Structure of Chitosan

**Figure 6:**
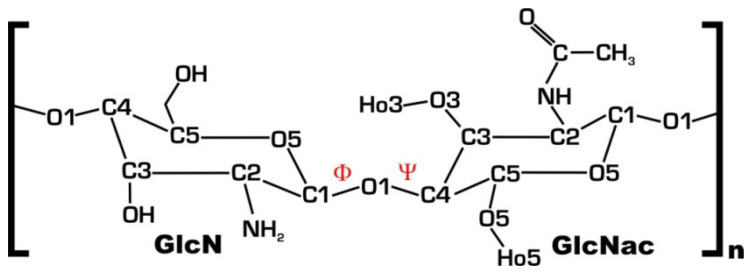
A disaccharide produced by 2-amino-2-deoxy-d-glucopyranose (GlcN) coupled to 2-acetamide-2-deoxy-d-glucopyranose (GlcNac) is seen schematically. In the scheme, dihedral angles are depicted in red and represent atoms O5-C1-O1-C4’ and C1-O1-C4’-C5’, respectively [47].

Chitosan is a semi-synthetic linear copolymer made up of an undetermined number of β-(1–4)-linked units of 2-acetamide-2-deoxy-β-d-glucopyranose (GlcNAc) and 2-amino-2-deoxy-β-d-glycopyranose (GlcN) units. (Figure 6) The C2-substituent in the sugar ring, an amino or acetamide group, distinguishes the two monomers. The alkaline deacetylation of chitin provides chitosan. In normal heterogeneous reactions, the deacetylation process is incomplete, resulting in a random distribution of GlcNAc and GlcN residues in the chitosan polymer. The average GlcNAc per 100 chitosan monomers in percentile unit indicates polymer acetylation. The solubility and conformation of the chitosan polymer depend on the level of acetylation [47].

Conventionally, we call it a chitosan if the chitin polymers’ acetylation level is below 50%. Chitosan’s solubility depends on the positively charged amino groups of glucosamine monomers, which make it cationic. The degree of deacetylation, also known as DDA, is a variable that indicates the proportion of glucosamine units present in the polymer. This proportion is one of the most important factors that determines the polymer’s physical and chemical characteristics [11,12,13].

Chitosan has the chemical formula (C_6_H_11_NO_4_)^n^, where “n” refers to the degree of polymerization and indicates the number of monomer units that are included in the chain of the polymer. The degree of polymerization can change, which leads to chitosan molecules with varying molecular weights and sizes. These factors, in turn, affect the solubility, viscosity, and biocompatibility of the chitosan molecules [10].

Chitosan’s molecular weight is a critical component that determines its qualities and performance in certain applications. Chitosan offers a wide range of molecular weights, which has a significant influence on its solubility, viscosity, biocompatibility, and overall usefulness. The average number of monomer units (glucosamine and *N*-acetylglucosamine) in chitosan’s polymer chain is measured by its molecular weight. It is commonly measured in daltons (Da) or kilodaltons (kDa). Chitosan has a wide range of molecular weights, ranging from a few thousand Da to several hundred thousand Da. This variation is caused by changes in the source material (e.g., crustacean shells, fungus), the degree of deacetylation (DDA), and the chitosan extraction techniques utilized [13,47,48].

Solubility is one of the most noticeable consequences of chitosan molecular weight. When opposed to high-molecular-weight chitosan, low-molecular-weight chitosan has smaller polymer chains and is more soluble in water and other aqueous solutions. The reduced size of the molecules allows for greater dispersion and dissolution in solvents, resulting in enhanced solubility. As a result, low-molecular-weight chitosan is frequently favored in applications requiring high solubility, such as medication administration and wound healing. Another attribute influenced by molecular weight is the viscosity of chitosan solutions. Because of the longer and bigger polymer chains, high-molecular-weight chitosan tends to generate more viscous solutions, resulting in a thicker and more gel-like consistency. This property is useful in applications requiring chitosan’s capacity to stick to surfaces and produce a barrier effect, such as gels, films, and coatings. Low-molecular-weight chitosan solutions are less viscous and more easily flowable [13,47,48].

Chitosan’s molecular weight also influences its biological activity and biocompatibility. High-molecular-weight chitosan has been proven in studies to have higher bio adhesive qualities, making it appropriate for use in tissue engineering, wound healing, and medication administration to mucosal surfaces. Furthermore, high-molecular-weight chitosan has been shown to induce physiological responses such as cell proliferation and tissue regeneration. Low-molecular-weight chitosan, on the other hand, is more easily absorbed in the gastrointestinal tract, making it a favored choice for oral medication administration. Its tiny size permits it to penetrate biological barriers more effectively, improving medication’s absorption and bioavailability [12].

Several studies have investigated the effects of chitosan on *S. mutans*, and this discussion will look at four of them [25,26,27,35]. Within dentistry, the effects of chitosan have been investigated in several areas, such as periodontics, endodontics, and orthodontics. These investigations have been performed on animal and human models, via in vitro studies [8,49,50,51].

#### 3.4.2. Chitosan Applications in Dentistry

Because of its molecular structure and capabilities, chitosan is an extremely useful component in a wide variety of dental applications. First, we must mention wound healing and tissue regeneration. Chitosan has been used in oral surgery and periodontal operations to accelerate wound healing and tissue regeneration. Chitosan has also been used in the treatment of periodontal disease. Because of its biocompatibility as well as its capacity to promote fibroblast proliferation and collagen production, it is a great choice for the promotion of tissue healing in the oral cavity [17,50,51]. For the formation of bio adhesive barriers, chitosan films can be applied to the oral mucosa, as well as the surfaces of the teeth. These films can protect oral wounds, hence reducing the risk of post-operative problems and increasing patient comfort [17,38].

Chitosan’s mucoadhesive qualities, which result from its interaction with mucin and allow it to be employed as a drug delivery system in the form of gels, films, or nanoparticles, must be mentioned when discussing oral drug delivery. These features enable chitosan to be used in drug delivery. These systems have the capability to deliver therapeutic substances locally, which enables them to treat oral infections, oral discomfort, and other dental disorders [12,13]. Because of its cationic property, chitosan is efficient against a wide variety of oral infections. It has been added to dental materials, such as mouthwashes, toothpaste, and other dental products, to impart antibacterial action and lower the probability of developing dental infections [8,13]. Dental composites and restorative materials have had chitosan nanoparticles added to them to increase their mechanical characteristics and prevent bacterial adherence. This has contributed to the durability of dental restorations [35,52].

Scaffolds made from chitosan have been utilized in the field of implant dentistry to stimulate bone growth around dental implants. Because of the material’s biocompatibility as well as its capacity to assist osteoblast proliferation and mineralization, it has the potential to become an important part of bone augmentation operations [40,49]. Products made from chitosan have been evaluated to see whether they can help manage periodontal disorders. Controlling the development of periodontal infections can be accomplished by utilizing these agents either in combination with conventional treatments or in sustained-release formulations [51].

#### 3.4.3. Analysis

Our study examined bacteria from human samples rather than a standard strain, which, although it increases the heterogeneity of the study due to the multiple serotypes in the oral cavity, is more useful in practice because it tests multiple strains of oral bacteria. All the articles we reviewed investigated antimicrobial activity against *S. mutans*. Saliva samples were taken in a similar, but not the same, way. Hayashi et al. in 2006 asked subjects to brush their teeth before. After this, pre-experimental saliva was collected after 5 min of wax mastication. Then, in the gum-chewing stage, the subjects chewed one piece of gum for 5 min, then rested for 5 min. This was repeated eight times, for a total of 80 min. Then, post-experimental saliva was collected at 0 min, 30 min and 60 min. Hayashi et al. (2007) used a similar method, but the gum-chewing stage only lasted for 50 min. Khamverdi et al. (2020) first collected pre-experimental saliva after 5 min of wax mastication, then in the gum-chewing stage, the subjects chewed for 5 min and rested for 5 min. This was repeated eight times, so all in all the gum-chewing stage lasted for 80 min (40 min chewing, 40 min rest) [25,26,27,35].

In each case, chitosan was placed in chewing gum, and the effect was tested after chewing the gum. A clear bacteriostatic/inhibitory effect was found in all cases. Chitosan has a minor cytotoxic effect, but this effect is outweighed by the positive ones [46]. Chitosan is digested by human colonic bacteria and porcine pancreatic enzymes [53]. In addition to its antimicrobial properties, the effects of chitosan on saliva volume and oral pH have also been studied. The pH is elevated, which is beneficial because it counteracts the acidic effect and makes the environment less cariogenic. Furthermore, the amount of saliva increases, which is also positive because more saliva means more buffer capacity, which also reduces the cariogenic environment [25,26,27,35].

**Figure 7:**
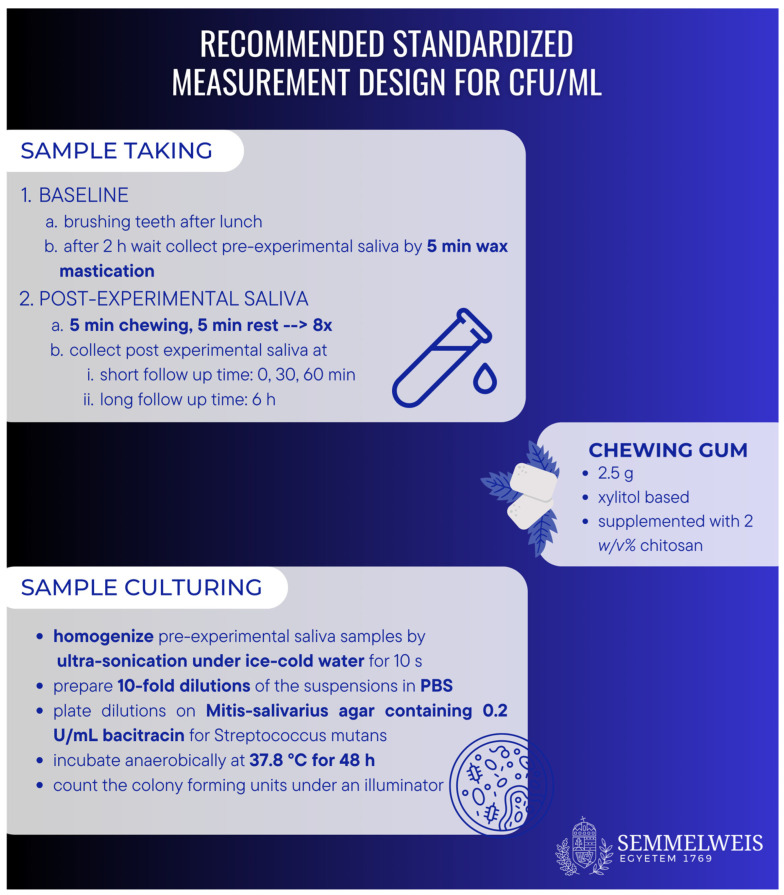
Recommended standardized measurement design. Sample taking: We should take a pre-experimental saliva sample for the baseline values and post-experimental saliva after 80 min of chewing chitosan-containing gum. For sample culturing, we used Mitis-salivarius agar with 0.2 U/mL bacitracin and incubated it for 48 h.

As more research in the future could help clinicians to derive clearer results, we recommend that future investigations utilize a standardized measuring design. This suggested design is shown on Figure 7.

The study by Hosseinpour et al. used a rat model with orthodontic appliances, and the results show that the chitosan nanoparticles had significant antibacterial effects against the biofilm of cariogenic bacteria, including *S. mutans* [35].

First, the rats had to become germ-free in the oral cavity, as their oral microbial flora differs from that of humans. Three human cariogenic bacteria, including *S. mutans* (ATCC 25175), were sampled. This article, in contrast to the studies mentioned earlier [25,26,27,35], investigated long-term effects. The efficacy of chitosan has also been demonstrated in this case, making it a great alternative for use in chewing gums and mouthwashes [35].

#### 3.4.4. Strengths and Limitations

In terms of the strengths of our analysis, we followed the pre-registered PROSPERO protocol. To achieve objectivity, both univariate and multivariate analyses were performed. This is the first meta-analysis on the use of chitosan in the dental field. All included articles were randomized–control trials. There were some limitations. For example, the baseline levels of bacteria varied widely between the populations of the three studies, and pooled estimates should be treated with caution. The estimation of standard deviations and correlation coefficients was also a limit. The presence of a risk of bias is of some concern in one of the domains. Articles assessed in the analysis only concerned studies conducted in two locations, with minimal participants, which impacts the meta-analysis and critical appraisal. Two studies were by the same author, and all papers examined Asian populations.

#### 3.4.5. Implications for Practice and Research

More clinical, in vitro, and animal studies are needed to provide a higher level of evidence regarding whether or not chitosan significantly reduces the number of *S. mutans*. The rapid translation of scientific findings into clinical applications is critical [54,55].

## 4. Conclusions

Based on our meta-analysis, chitosan has some antibacterial properties when utilized as an ingredient in chewing gum, but further research is required to obtain more significant findings. Our recommended measurement design provides guidance for further studies, and will yield more homogeneous data. 

## Figures and Tables

**Table 1:** Basic characteristics of the studies included.

First Author	Year of Publication	DOI	Study Design	Country	Mean Age	No. of Patients in Control	No. of Patients in Intervention	Intervention	Control	Ref.
Khamverdi	2020	10.1080/00016357.2020.1836392	RCT	Iran	22.74	18	18	chitosan	placebo	[27]
Hayashi	2007	10.1016/j.jdent.2007.08.004	RCT	Japan	32.8	6	6	chitosan	placebo	[25]
Hayashi	2006	10.1016/j.archoralbio.2006.10.004	RCT	Japan	23.7	50	50	chitosan	placebo	[26]

## Data Availability

Data are contained within the article and Supplementary Materials.

## Supplementary Materials

The following supporting information can be downloaded at: https://www.mdpi.com/article/10.3390/ijms242015270/s1.

## References

[1] Bae K., Jun E.J., Lee S.M., Paik D.I., Kim J.B. (2006). Effect of water-soluble reduced chitosan on *Streptococcus mutans*, plaque regrowth and biofilm vitality. Clin. Oral. Investig..

[2] Fujiwara M., Hayashi Y., Ohara N. (2004). Inhibitory effect of water-soluble chitosan on growth of *Streptococcus mutans*. New Microbiol..

[3] Helander I.M., Latva-Kala K., Lounatmaa K. (1998). Permeabilizing action of polyethyleneimine on Salmonella typhimurium involves disruption of the outer membrane and interactions with lipopolysaccharide. Microbiology.

[4] Sano H., Shibasaki K.-I., Matsukubo T., Takaesu Y. (2003). Effect of chitosan rinsing on reduction of dental plaque formation. Bull. Tokyo Dent. Coll..

[5] Englander H.R., Keyes P.H. (1971). Control of *Streptococcus* mutans, plaque, and dental caries in hamsters with topically applied vancomycin. Arch. Oral Biol..

[6] Qiu W., Zhou Y., Li Z., Huang T., Xiao Y., Cheng L., Peng X., Zhang L., Ren B. (2020). Application of Antibiotics/Antimicrobial Agents on Dental Caries. BioMed Res. Int..

[7] DePaola P.F., Jordan H.V., Soparkar P.M. (1977). Inhibition of dental caries in school children by topically applied vancomycin. Arch Oral Biol..

[8] Yilmaz Atay H. (2020). Antibacterial Activity of Chitosan-Based Systems. Functional Chitosan: Drug Delivery and Biomedical Applications.

[9] Wei X.Y., Xia W., Zhou T. (2022). Antibacterial activity and action mechanism of a novel chitosan oligosaccharide derivative against dominant spoilage bacteria isolated from shrimp Penaeus vannamei. Lett. Appl. Microbiol..

[10] Khajavian M., Vatanpour V., Castro-Muñoz R., Boczkaj G. (2022). Chitin and derivative chitosan-based structures—Preparation strategies aided by deep eutectic solvents: A review. Carbohydr. Polym..

[11] Riaz Rajoka M.S., Zhao L., Mehwish H.M., Wu Y., Mahmood S. (2019). Chitosan and its derivatives: Synthesis, biotechnological applications, and future challenges. Appl. Microbiol. Biotechnol..

[12] Satitsri S., Muanprasat C. (2020). Chitin and Chitosan Derivatives as Biomaterial Resources for Biological and Biomedical Applications. Molecules.

[13] Younes I., Rinaudo M. (2015). Chitin and chitosan preparation from marine sources. Structure, properties and applications. Mar. Drugs.

[14] Jensen D.M., Han P., Mangala L.S., Lopez-Berestein G., Sood A.K., Liu J., Kriegel A.J., Usa K., Widlansky M.E., Liang M. (2022). Broad-acting therapeutic effects of miR-29b-chitosan on hypertension and diabetic complications. Mol. Ther..

[15] Mehrabi M., Montazeri H., Mohamadpour Dounighi N., Rashti A., Vakili-Ghartavol R. (2018). Chitosan-based Nanoparticles in Mucosal Vaccine Delivery. Arch Razi. Inst..

[16] Lütjohann D., Marinova M., Wolter K., Willinek W., Bitterlich N., Coenen M., Coch C., Stellaard F. (2018). Influence of Chitosan Treatment on Surrogate Serum Markers of Cholesterol Metabolism in Obese Subjects. Nutrients.

[17] Patrulea V., Ostafe V., Borchard G., Jordan O. (2015). Chitosan as a starting material for wound healing applications. Eur. J. Pharm. Biopharm..

[18] Struzycka I. (2014). The oral microbiome in dental caries. Pol. J. Microbiol..

[19] Lemos J.A., Palmer S.R., Zeng L., Wen Z.T., Kajfasz J.K., Freires I.A., Abranches J., Brady L.J. (2019). The Biology of *Streptococcus mutans*. Microbiol. Spectr..

[20] Nakano K., Ooshima T. (2009). Serotype classification of *Streptococcus mutans* and its detection outside the oral cavity. Future Microbiol..

[21] Figuero E., Nóbrega D.F., García-Gargallo M., Tenuta L.M., Herrera D., Carvalho J.C. (2017). Mechanical and chemical plaque control in the simultaneous management of gingivitis and caries: A systematic review. J. Clin. Periodontol..

[22] Oztaş N., Bodur H., Olmez A., Berkkan A., Cula S. (2004). The efficacy of a fluoride chewing gum on salivary fluoride concentration and plaque pH in children. J. Dent..

[23] Imfeld T. (1999). Chewing gum--facts and fiction: A review of gum-chewing and oral health. Crit. Rev. Oral Biol. Med..

[24] Dodds M.W., Chidichimo D., Haas M.S. (2012). Delivery of active agents from chewing gum for improved remineralization. Adv. Dent. Res..

[25] Hayashi Y., Ohara N., Ganno T., Ishizaki H., Yanagiguchi K. (2007). Chitosan-containing gum chewing accelerates antibacterial effect with an increase in salivary secretion. J. Dent..

[26] Hayashi Y., Ohara N., Ganno T., Yamaguchi K., Ishizaki T., Nakamura T., Sato M. (2007). Chewing chitosan-containing gum effectively inhibits the growth of cariogenic bacteria. Arch. Oral Biol..

[27] Khamverdi Z., Farhadian F., Khazaei S., Adabi M. (2021). Efficacy of chitosan-based chewing gum on reducing salivary S. mutans counts and salivary pH: A randomised clinical trial. Acta Odontol. Scand..

[28] Beck B.H., Yildirim-Aksoy M., Shoemaker C.A., Fuller S.A., Peatman E. (2019). Antimicrobial activity of the biopolymer chitosan against *Streptococcus iniae*. J. Fish Dis..

[29] Matica M.A., Aachmann F.L., Tøndervik A., Sletta H., Ostafe V. (2019). Chitosan as a Wound Dressing Starting Material: Antimicrobial Properties and Mode of Action. Int. J. Mol. Sci..

[30] Moraru C., Mincea M.M., Frandes M., Timar B., Ostafe V. (2018). A Meta-Analysis on Randomised Controlled Clinical Trials Evaluating the Effect of the Dietary Supplement Chitosan on Weight Loss, Lipid Parameters and Blood Pressure. Medicina.

[31] Ji H., Wang J., Chen F., Fan N., Wang X., Xiao Z., Wang Z. (2022). Meta-analysis of chitosan-mediated effects on plant defense against oxidative stress. Sci. Total Environ..

[32] Harahap R.P., Suharti S., Ridla M., Laconi E.B., Nahrowi N., Irawan A., Kondo M., Obitsu T., Jayanegara A. (2022). Meta-analysis of dietary chitosan effects on performance, nutrient utilization, and product characteristics of ruminants. Anim. Sci. J..

[33] Guo W., Yi L., Zhou B., Li M. (2020). Chitosan modifies glycemic levels in people with metabolic syndrome and related disorders: Meta-analysis with trial sequential analysis. Nutr. J..

[34] Higgins J.P.T., Chandler J., Cumpston M., Li T., Page M.J., Welch V.A. Cochrane Handbook for Systematic Reviews of Interventions Version 6.3 (Updated February 2022). http://www.training.cochrane.org/handbook.

[35] Hosseinpour Nader A., Sodagar A., Akhavan A., Pourhajibagher M., Bahador A. (2020). Antibacterial Effects of Orthodontic Primer Harboring Chitosan Nanoparticles against the Multispecies Biofilm of Cariogenic Bacteria in a Rat Model. Folia Med..

[36] Dai T., Tanaka M., Huang Y.Y., Hamblin M.R. (2011). Chitosan preparations for wounds and burns: Antimicrobial and wound-healing effects. Expert. Rev. Anti. Infect. Ther..

[37] Elviri L., Bianchera A., Bergonzi C., Bettini R. (2017). Controlled local drug delivery strategies from chitosan hydrogels for wound healing. Expert Opin. Drug Deliv..

[38] Vigani B., Rossi S., Sandri G., Bonferoni M.C., Caramella C.M., Ferrari F. (2019). Hyaluronic acid and chitosan-based nanosystems: A new dressing generation for wound care. Expert. Opin. Drug. Deliv..

[39] Matalqah S.M., Aiedeh K., Mhaidat N.M., Alzoubi K.H., Bustanji Y., Hamad I. (2020). Chitosan Nanoparticles as a Novel Drug Delivery System: A Review Article. Curr. Drug Targets.

[40] Bharathi R., Ganesh S.S., Harini G., Vatsala K., Anushikaa R., Aravind S., Abinaya S., Selvamurugan N. (2022). Chitosan-based scaffolds as drug delivery systems in bone tissue engineering. Int. J. Biol. Macromol..

[41] Ahsan S.M., Thomas M., Reddy K.K., Sooraparaju S.G., Asthana A., Bhatnagar I. (2018). Chitosan as biomaterial in drug delivery and tissue engineering. Int. J. Biol. Macromol..

[42] Aguilar A., Zein N., Harmouch E., Hafdi B., Bornert F., Offner D., Clauss F., Fioretti F., Huck O., Benkirane-Jessel N. (2019). Application of Chitosan in Bone and Dental Engineering. Molecules.

[43] Upadhyaya L., Singh J., Agarwal V., Tewari R.P. (2014). The implications of recent advances in carboxymethyl chitosan based targeted drug delivery and tissue engineering applications. J. Control Release.

[44] Pandiyan I., Rathinavelu P.K., Arumugham M.I., Srisakthi D., Balasubramaniam A. (2022). Efficacy of Chitosan and Chlorhexidine Mouthwash on Dental Plaque and Gingival Inflammation: A Systematic Review. Cureus.

[45] Camacho-Alonso F., Julián-Belmonte E., Chiva-García F., Martínez-Beneyto Y. (2017). Bactericidal Efficacy of Photodynamic Therapy and Chitosan in Root Canals Experimentally Infected with *Enterococcus faecalis*: An In Vitro Study. Photomed. Laser Surg..

[46] Yaroslavov A.A., Efimova A.A., Krasnikov E.A., Trosheva K.S., Popov A.S., Melik-Nubarov N.S., Krivtsov G.G. (2021). Chitosan-based multi-liposomal complexes: Synthesis, biodegradability and cytotoxicity. Int. J. Biol. Macromol..

[47] Richard A.C., Thereza A.S., Victor H.R., Frederico J.S.P., Eduardo F.F., Roberto D.L., Desiree Nedra K. (2012). The Molecular Structure and Conformational Dynamics of Chitosan Polymers: An Integrated Perspective from Experiments and Computational Simulations. The Complex World of Polysaccharides.

[48] Chen S., Ma X., Han Y., Wei Y., Guo Q., Yang S., Zhang Y., Liao W., Gao Y. (2020). Effect of chitosan molecular weight on zein-chitosan nanocomplexes: Formation, characterization, and the delivery of quercetagetin. Int. J. Biol. Macromol..

[49] Anggani H.S., Perdana R.G., Siregar E., Bachtiar E.W. (2021). The effect of coating chitosan on Porphyromonas gingivalis biofilm formation in the surface of orthodontic mini-implant. J. Adv. Pharm. Technol. Res..

[50] Xu C., Lei C., Meng L., Wang C., Song Y. (2012). Chitosan as a barrier membrane material in periodontal tissue regeneration. J. Biomed. Mater. Res. B Appl. Biomater..

[51] Thangavelu A., Stelin K.S., Vannala V., Mahabob N., Hayyan F.M.B., Sundaram R. (2021). An Overview of Chitosan and Its Role in Periodontics. J. Pharm. Bioallied. Sci..

[52] Tanaka C.B., Lopes D.P., Kikuchi L.N.T., Moreira M.S., Catalani L.H., Braga R.R., Kruzic J.J., Gonçalves F. (2020). Development of novel dental restorative composites with dibasic calcium phosphate loaded chitosan fillers. Dent. Mater..

[53] Baldrick P. (2010). The safety of chitosan as a pharmaceutical excipient. Regul. Toxicol. Pharmacol..

[54] Hegyi P., Erőss B., Izbéki F., Párniczky A., Szentesi A. (2021). Accelerating the translational medicine cycle: The Academia Europaea pilot. Nat. Med..

[55] Hegyi P., Petersen O.H., Holgate S., Erőss B., Garami A., Szakács Z., Dobszai D., Balaskó M., Kemény L., Peng S. (2020). Academia Europaea Position Paper on Translational Medicine: The Cycle Model for Translating Scientific Results into Community Benefits. J. Clin. Med..

