# Supplementary material for: Effect of Chitosan on the Number of Streptococcus mutans in Saliva: A Meta-Analysis and Systematic Review
Source: Int J Mol Sci. 2023 Oct 17;24(20):15270. doi: 10.3390/ijms242015270 (PMC10607225; doi:10.3390/ijms242015270)
Supplement: Supplementary file 1 [file ijms-24-15270-s001.zip › Table S1.pdf]

Table S1. Evidence synthesis: GRADE-PRO

Certainty assessment							№ of patients		Effect		Certainty	Importance
№ of studies	Study design	Risk of bias	Inconsistency	Indirectness	Imprecision	Other considerations	chitosan	placebo	Relative (95% CI)	Absolute (95% CI)		
CFU/mL (assessed with: illuminator; Scale from: 0 to 60)												
3	randomised trials	not serious	not serious	not serious	not serious		74	74	-	MD **0** (12.5×10^5 lower to 30.6×10^4 higher)	-	CRITICAL
CFU/mL baseline (assessed with: illuminator; Scale from: 0 to 0)												
3	randomised trials	not serious	not serious	not serious	not serious		74	74	-	**0** (1250000 lower to 306000 higher)	-	CRITICAL

CI: confidence interval; **MD:** mean difference
